# Genome Sequence of *Klebsiella pneumoniae* Bacteriophage PMBT1 Isolated from Raw Sewage

**DOI:** 10.1128/genomeA.00914-16

**Published:** 2017-02-23

**Authors:** Sabrina Koberg, Erik Brinks, Gregor Fiedler, Christina Hüsing, Gyu-Sung Cho, Marc P. Hoeppner, Knut J. Heller, Horst Neve, Charles M. A. P. Franz

**Affiliations:** a Department of Microbiology and Biotechnology, Max Rubner-Institut (Federal Research Institute of Nutrition and Food), Kiel, Germany; b Institute of Clinical Molecular Biology (IKMB), Kiel University, Kiel, Germany

## Abstract

A bacteriophage virulent for extended-spectrum beta-lactamase (ESBL)-producing *Klebsiella pneumoniae* strain 182 was isolated from sewage. The double-stranded DNA (dsDNA) genome showed high similarity to the genomes of other *Klebsiella pneumoniae* phages. It comprises 175,206 bp with a mol% G+C content of 41.9 and contains 276 putative open reading frames (ORFs) and one tRNA.

## GENOME ANNOUNCEMENT

Multidrug-resistant Gram-negative bacteria belonging to the *Klebsiella* species are currently recognized as the most frequent cause of bacterial hospital outbreaks (1). This is mainly due to the emergence of extended-spectrum beta-lactamase (ESBL)-producing strains (1, 2). Infections caused by *Klebsiella* spp. include pneumonia, urinary tract infections, pyogenic liver abscesses, bacteremia, and septic shock (3, 4). The genes responsible for beta-lactam resistance are carried on and transferred by plasmids among different species (5). They encode extended-spectrum beta-lactamases, which hydrolyze penicillins, third-generation cephalosporins, and monobactams (4). The extensive spread of multidrug-resistant strains, which encode not only ESBLs but also carbapenemases and diverse aminoglycoside-inactivating enzymes, has renewed global interest in fighting these pathogens (6). In view of the problem of development of multidrug-resistant strains, bacteriophages are being explored as an alternative treatment option (6).

Currently, complete genome sequences of a total of 32 *Klebsiella* phages have been deposited in GenBank/RefSeq (NCBI). They all belong to the double-stranded DNA (dsDNA) *Caudovirales*, comprising the families *Myoviridae*, *Siphoviridae*, and *Podoviridae* (7). In this study, we report on the genome sequence of the virulent phage PMBT1 isolated from wastewater of a municipal sewage plant located close to Kiel (northern Germany). The ESBL-producing *K. pneumoniae* strain 182, used as the host bacterium, was obtained from the culture collection of the Central Medical Service Institute of German Armed Forces Kiel-Kronshagen. Transmission electron microscopy revealed a phage morphotype with a prolate head (80 by 110 nm) and a 116-nm-long contractile tail. These characteristics assigned phage PMBT1 to the *Myoviridae* family.

DNA was isolated from high-titer phage lysates using the phage DNA isolation kit (Biocat, Heidelberg, Germany). The Nextera XT DNA library preparation kit and the MiSeq reagent kit version 3 were used for genome sequencing, according to the manufacturer’s instructions (Illumina, Munich, Germany), on the MiSeq platform, which produced 414,870 reads. A total of 400,970 paired-end reads were *de novo* assembled in a contig, with a total length of 175,206 bp, using Geneious 9.1.2 (8). The sequence has a mol% G+C content of 41.9. Automated annotation was made with RAST (9), followed by manual curation, resulting in 276 open reading frames (ORFs) with a start and stop codon, as well as a ribosomal-binding site. The smallest ORF encodes a putative protein with 27 amino acids. Genomic analysis revealed no lysogeny-related genes, thus confirming the virulent nature of PMBT1. A T4-like tail-sheath gene was found. The data showed that *K. pneumoniae* phage PMBT1 exhibits high similarity with T4-like *Klebsiella* phages KP15 (95.5%) and KP27 (94%) (10), with *Enterobacter* phage phiEap-3 (accession no. KT321315), and with pseudo-T-even phages Matisse (11) and Miro (12), respectively. One tRNA was identified using tRNAscan-SE version 1.21 (http://lowelab.ucsc.edu/tRNAscan-SE/).

### Accession number(s).

The complete genome sequence of *K. pneumoniae* phage PMBT1 generated in this project was deposited in the European Nucleotide Archive (ENA) under the accession no. LT607758.
